# Analysis of Public Oral Toxicity Data from REACH Registrations 2008–2014

**DOI:** 10.14573/altex.1510054

**Published:** 2016-02-11

**Authors:** Thomas Luechtefeld, Alexandra Maertens, Daniel P. Russo, Costanza Rovida, Hao Zhu, Thomas Hartung

**Affiliations:** 1 Center for Alternatives to Animal Testing (CAAT), Johns Hopkins Bloomberg School of Public Health, Environmental Health Sciences, Baltimore, MD, USA; 2 The Rutgers Center for Computational & Integrative Biology, Rutgers University at Camden, NJ, USA; 3 Department of Chemistry, Rutgers University at Camden, NJ, USA; 4 CAAT-Europe, University of Konstanz, Konstanz, Germany

**Keywords:** regulatory toxicology, chemical safety, LD_50_, acute toxicity, computational toxicology

## Abstract

The European Chemicals Agency, ECHA, made available a total of 13,832 oral toxicity studies for 8,568 substances up to December 2014. 75% of studies were from the retired OECD Test Guideline 401 (11% TG 420, 11% TG 423 and 1.5% TG 425). Concordance across guidelines, evaluated by comparing LD_50_ values ≥ 2,000 or < 2,000 mg/ kg bodyweight from chemicals tested multiple times between different guidelines, was at least 75% and for their own repetition more than 90%.

In 2009, Bulgheroni et al. created a simple model for predicting acute oral toxicity using no observed adverse effect levels (NOAEL) from 28-day repeated dose toxicity studies in rats. This was reproduced here for 1,625 substances. In 2014, Taylor et al. suggested no added value of the 90-day repeated dose oral toxicity test given the availability of a low 28-day study with some constraints. We confirm that the 28-day NOAEL is predictive (albeit imperfectly) of 90-day NOAELs, however, the suggested constraints did not affect predictivity.

1,059 substances with acute oral toxicity data (268 positives, 791 negatives, all Klimisch score 1) were used for modeling: The Chemical Development Kit was used to generate 27 molecular descriptors and a similarity-informed multilayer perceptron showing 71% sensitivity and 72% specificity. Additionally, the k-nearest neighbors (KNN) algorithm indicated that similarity-based approaches alone may be poor predictors of acute oral toxicity, but can be used to inform the multilayer perceptron model, where this was the feature with the highest information value.

## 1 Introduction

Acute oral toxicity data is a key component of classification and labeling as well as hazard assessment for industrial substances and is a requirement in diverse regulatory testing regimes. The first acute oral toxicity test was created by the British pharmacologist J. W. Trevan in 1927. It used to involve up to 150 animals until the OECD Test Guideline (TG) 401 standardized it to 45 animals in 1981.

The usefulness of the OECD TG 401 test has often been challenged. Gerhard Zbinden in the 1980s described the standard *in vivo* test as little more than “a ritual mass execution of animals”^1^. However, the classification derived from the test results impacts on worker protection and transport safety regulations. For this reason it is one of the most common assessments performed and was responsible for about one third of the animals used in toxicology in the 1990s^2^. The abolition of the original OECD TG 401 and its replacement by tiered testing strategies, which effectively reduced the number of animals further from 45 to 8–12, is probably one of the most animal-saving reduction methods in toxicology. REACH has changed the testing demands and patterns as for the first time high-production volume substances with complex test requirements came to the foreground (Rovida and Hartung, 2009; Hartung and Rovida, 2009). Still, all REACH substances require acute toxicity data by 2018 (Hartung, 2010).

The tiered testing methods replacing OECD TG 401 introduced in 2002 were the Fixed Dose Procedure (OECD TG 420), Acute Toxic Class Method (OECD TG 423), and Up and Down Dosing (OECD TG 425). While OECD TG 423 primarily identifies classification limits, the reported results of tests performed according to this guideline within ECHA dossiers include LD_50_ assessments. Of course, each of these tests extrapolates from a fairly limited dosing regime to estimate LD_50_ – with likely different levels of accuracy and biases. This complicates any attempt to build a predictive model.

Importantly, the majority of industrial substances are of low oral toxicity. It was shown earlier (Bulgheroni et al., 2009) that the European New Chemicals Database (NCD) in 2008 included 4,773 substances of which 4,219 included oral toxicity data: 70.2% were Category 5 (LD_50_ 2,000 – 5,000 mg/kg bodyweight (b.w.)) and 16.7% were “Not classified” LD_50_ > 5,000 mg/kg b.w.). This means that the most important questions are whether a substance falls into these categories and whether potency testing needs to be done for highly toxic substances with LD_50_ below 2,000 mg/kg. However, the NCD contains predominantly low-production volume specialty substances and therefore might not reflect the high-production volume and special concern substances (carcinogenic, mutagenic, reproductive toxin) of the early REACH deadlines analyzed here.

Oral toxicity is also assessed in repeated-dose toxicity studies in rodents: the (subacute) 28-day Oral Toxicity Study in Rodents (OECD TG 407) and the (subchronic) 90-Day Oral Toxicity Study in Rodents (OECD TG 408) are commonly employed as cornerstones for risk assessments. These assays include complex assessments of many organ toxicities by pathohistology, clinical chemistry and others. The studies yield information on general characteristics of the toxicity, the target organs of toxicity, the dose-response (curve) for each toxicity endpoint, responses to toxic metabolites formed in the organism, delayed responses, cumulative effects, the margin between toxic/non-toxic dose, information on reversibility/irreversibility of the effect, and NOAEL (No Observed Adverse Effect Level), NOEL (No Observed Effect Level) for toxicity. In the REACH legislation (Regulation (EC) No 1907/2006), TG 407 is required for substances with more than 10 tons and both TG 407 and TG 408 must be done for substances with more than 100 tons of production or marketing volume per year. Here, only the derived NOEL will be analyzed.

ECHA’s public database of REACH registration dossiers offers a large toxicology dataset useful for modeling toxicological studies. Here, we examine two hypotheses concerning oral toxicity – that a 28-day oral toxicity test can predict the 90-day oral toxicity test, and that a 28-day by iteratively training test can predict acute toxicity. Finally, we demonstrate that for the ECHA dataset there is a clear clustering of chemically similar, acutely toxic substances and evidence that both QSARs and read-across can accurately assess oral acute toxicity provided an appropriate applicability domain has been defined.

## 2 Material and methods

### 2.1 LD_50_ oral toxicity data

Oral toxicity data was extracted from studies in ECHA dossiers as described in Luechtefeld et al. (2016, this issue). ECHA studies for experimental oral toxicity reporting an LD_50_ value in mg/ kg body weight were aggregated into a dataset of 13,832 studies over 8,568 substances. A substance LD_50_ was defined as the average substance LD_50_ reported in studies meeting stated criteria (Klimisch score/guideline number).

### 2.2 Cheminformatic methods

Chemical similarity and molecular descriptors were derived with the Chemistry Development Kit (CDK) version 1.5.11 (Stein-beck et al., 2003) for substances in ECHA disseminated studies. CDK provides methods for generating chemical structural fingerprints as well as molecular descriptors. 3,122 substances that could be mapped from REACH to SMILES were analyzed using CDK.

Similarity between substances was calculated via the Tanimo-to distance, a binary based similarity measure (Lourenço et al., 2004) as the number of shared substructures divided by the total number of substructures. We used supervised and instance-based learning methods (Hall et al., 2009; Aha et al., 1991) to predict oral toxicity from chemical descriptors and chemical similarity.

### 2.3 Machine learning

K-nearest neighbors (KNN) was implemented for this study. We predict a chemical as an oral toxicant (LD_50_ < 2,000 mg/ kg) if the majority of its 5 closest neighbors are oral toxicants (LD_50_ < 2,000 mg/kg) where a chemical is considered a neighbor if its Tanimoto similarity is greater than or equal to 70%.

Multilayer perceptron and decision tree supervised learners were trained using the 29 molecular descriptors available via CDK. The Weka machine learning toolkit implementation of multilayer perceptron was used with default parameters (Hall et al., 2009). Weka version 3.6.12 was used for training and testing of instance-based and supervised learning methods.

KNN was fused with multilayer perceptron to create a combined supervised learner by using the output of the KNN model as a feature in the multilayer perceptron.

### 2.4 Feature importance

Two approaches were used to evaluate feature importance. For both approaches we split the dataset into 100 sub-datasets of equal size with balanced toxicant/non-toxicant frequency.

In the first approach we evaluate the information gain of each feature across the sub datasets. In the second approach we evaluate a classifier (decision tree classifier) by iteratively training on a training set comprised of 99 datasets and testing on the remaining one dataset. Features were then removed and impact on accuracy was ascertained. Features whose removal result in greater accuracy reduction show more promise of being part of an effective set of features.

The first approach we refer to as the Ranker and the second approach as the WrapperEval; these names are derived from their implementation in the machine learning toolkit (Hall et al., 2009).

## 3 Results

### 3.1 OECD guideline prevalence and LD_50_ toxicity results

Within the ECHA database, results and discussions data give information on effective doses, effect levels (NOAELS, LELs, etc.), observed mortality, observed clinical signs, physical chemical properties and more. Unfortunately, the results data in the ECHA disseminated database does not follow a standard data format. Many studies record results in natural language (such as “sensitizing” or “no observed dose for acute toxicity”). These limitations should be considered when evaluating the reliability of our ECHA dossier results. Despite the lack of a standardized format and the use of natural language, it is still relatively simple to automatically group together studies that share a single OECD TG or are related to a common endpoint.

**Fig. 1:**
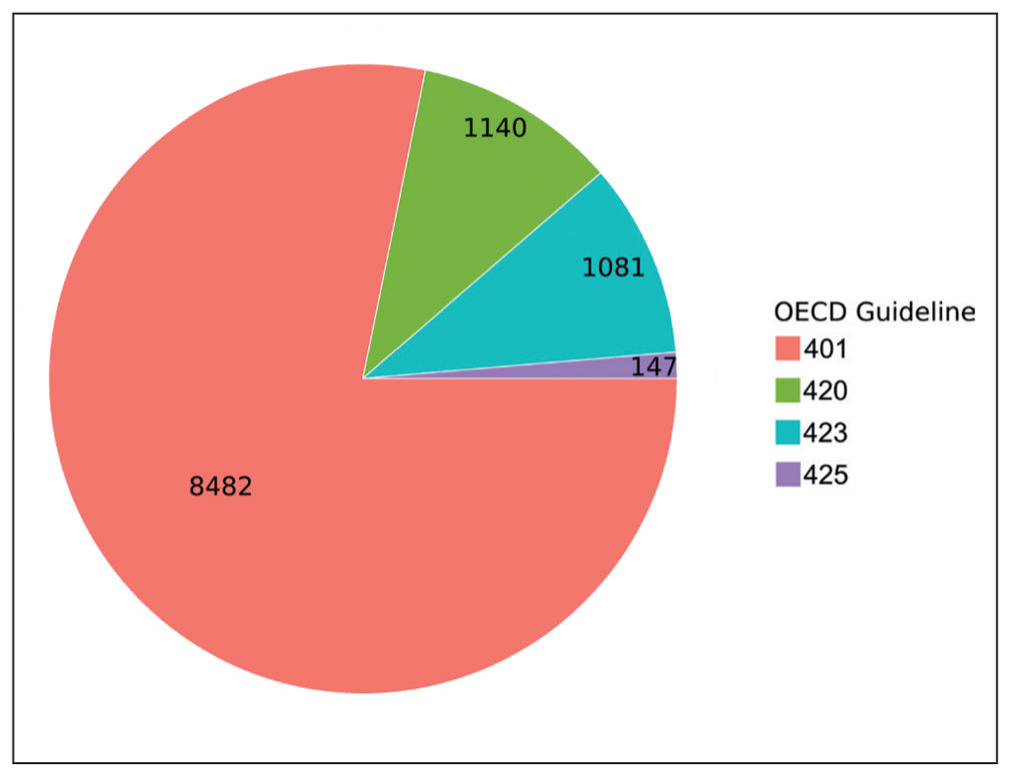
Number of substances with studies for each of the acute oral toxicity OECD guidelines The Limit Test (OECD TG 401, now deleted) was performed on 8,482 substances, the Fixed Dose Procedure (OECD TG 420) on 1,140 substances, the Chemical Classification Test (OECD TG 423) on 1,081 substances, and Up and Down Dosing (OECD TG 425) on 147 substances.

**Fig. 2:**
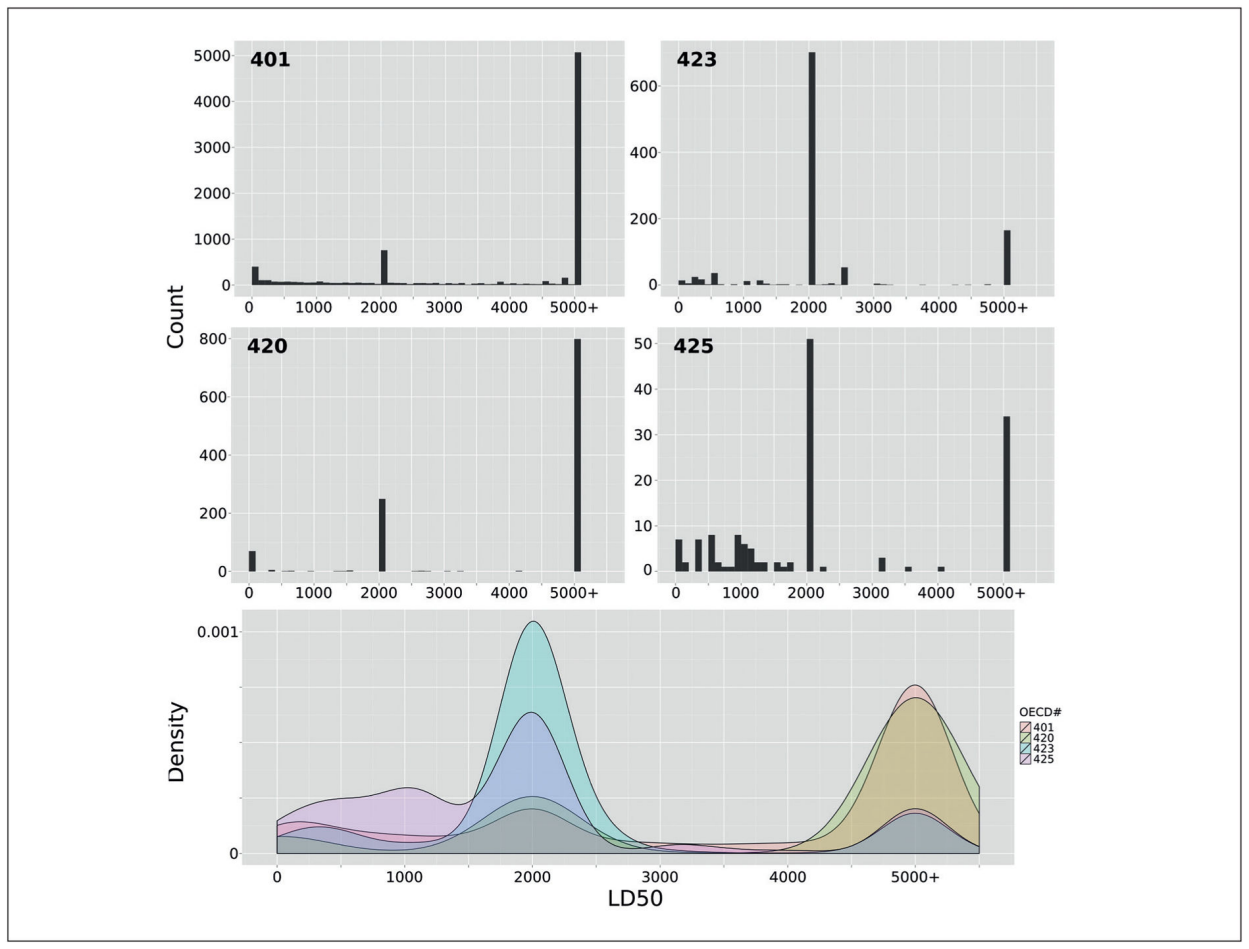
Histograms for use of OECD TG 401, 420, 423, and 425 in registrations of acute oral toxicant OECD TG 401, 423, 420, and 425 Y-axes are not equivalent. The x-axis represents LD_50_ for each OECD guideline. Density plot with overlapping densities between 0 and 5,000 mg/kg dosage. Notice the LD_50_ clustering around 2,000 and 5,000 mg/kg dosage; this is due to dosing schemes.

As our values were extrapolated according to the main OECD TG methodologies for acute toxicity, we were able to examine both the use of the various testing guidelines and the resulting distributions of LD_50_ values. As can be seen in Figure 1, the overwhelming number of studies were performed according to OECD TG 401 (OECD, 1987), while OECD TG 425 (Up and Down Procedure) was the least common. Other studies have shown strong agreement in terms of hazard classification among OECD TG data^3^ (Lipnick et al., 1995). However, a look at the LD_50_ distributions points to an interesting difference between OECD test guidelines in the distribution of calculated LD_50_ values. Figure 2 shows a substantial skew in the distribution for OECD TG 423 and 425 towards lower doses, which is likely the result of the dosing regime and extrapolation method – an important difference between methodologies, which is prudent to keep in mind when predicting LD_50_.

Table 1 gives the number of substances with labels H300 through H305. Substances given a label are reported here as “positive” and otherwise as “conclusive but not sufficient evidence for classification”, “data lacking” and “inconclusive”. These hazards cover harm caused via oral intake of substances. 0.6% of substances were “Fatal if swallowed” (H300), 3.9% “Toxic if swallowed” (H301), 18.7% “Harmful if swallowed” (H302) and 0.4% “May be harmful if swallowed” (H303), which leaves 76.4% that were not orally toxic.

We were able to extend the previous studies of concordance across guidelines by evaluating the consistency of results for substances tested under more than one TG. In this analysis we consider guidelines to be in agreement when both either report LD_50_ ≥ 2,000 mg/kg b.w. or both report LD_50_ < 2,000. Table 2 shows percent agreement for guidelines averaged over substances with the number of substances with data for both TGs in parentheses. All TGs show > 90% self-consistency. With the exception of the TG 401 versus 423 comparison, all guidelines show at least 80% inter-guideline consistency; our results are thus within the same range as previously reported (Lipnick et al., 1995).

### 3.2 Testing mutual information

Previous attempts to evaluate mutual information between different toxicological tests have involved tedious and error-prone manual curation of both ECHA and other toxicological studies (Taylor et al., 2014; Janer et al., 2007; Dang et al., 2009). Manual curation limits both the possible size of analyzed datasets and the applicability of the results to larger, more diverse chemical sets due to the inherent subjectivity of manual human analysis as well as considerably greater required resources.

Fortunately, the REACH-extracted data allow for a preliminary evaluation of two well-known oral toxicity redundancy claims on a dataset considerably larger than those initially used. In 2009, Bulgheroni et al. evaluated whether acute oral toxicity could be predicted (and thus made redundant) by repeat-dose 28 day toxicity data. Bulgheroni et al. used authorized access to the NCD (Bulgheroni et al., 2009). In 2014, Taylor et al. published an evaluation of the added value of the 90-day repeated dose oral toxicity test given the availability of a “sub-acute toxicity profile” (Taylor et al., 2014).

Bulgheroni’s analysis identified 1,791 substances with acute oral LD_50_ and 28-day oral NOAEL data from the NCD (Bulgheroni et al., 2009). The evaluation indicated that a 200 mg/kg body weight (b.w.) threshold for the 28-day NOAEL was strongly predictive for negative acute oral toxicity (LD_50_ > 2,000 mg/kg b.w.) with a negative predictive value of 97% and a positive predictive value of 27%. Consequently, the authors suggested a testing strategy whereby a > 2,000 mg/kg b.w. LD_50_ estimation could be made given a NOAEL value > 200 mg/kg b.w. (Bulgheroni et al. 2009). Collation of ECHA disseminated acute toxicity oral LD_50_ results (OECD TG 401, 423, 420 and 425) and 28-day NOAELs (OECD 407) allows for the evaluation of this testing strategy on the extracted substances. Table 3 gives the Bulgheroni threshold counts, i.e., NOAELs > 200 mg/kg and < 200 mg/kg, on 1,625 REACH extracted substances with key study LD_50_ values (white) and on 5,270 substances with any study LD_50_ data (including key studies, ReadAcross and non-key studies) (grey).

We find the proposed 200 mg/kg b.w. NOAEL threshold to have an 89.9% negative predictive value for key studies, meaning that a substance with > 200 mg/kg b.w. NOAEL will also have LD_50_ > 2,000. When the 200 mg/kg b.w. NOAEL threshold is considered for all studies, it has a negative predictive value of 94.5%. Key studies show a positive predictive value of 38.5%. All studies show a positive predictive value of 33.7%. These results are in close agreement with the statistics published by Bulgheroni et al. (2009), and indicate that the results are largely robust for different chemical types – lack of toxicity in a subchronic study is broadly indicative of lack of acute toxicity. If a 28-day NOAEL of 200 mg/kg per day had been used to rule out acute toxicity, then 928 previous acute oral toxicity studies could have been avoided. It should be noted that a method will be required to set dose levels for regulatory studies such as OECD TG 407 if acute toxicity testing is replaced in line with these suggestions.

**Fig 3:**
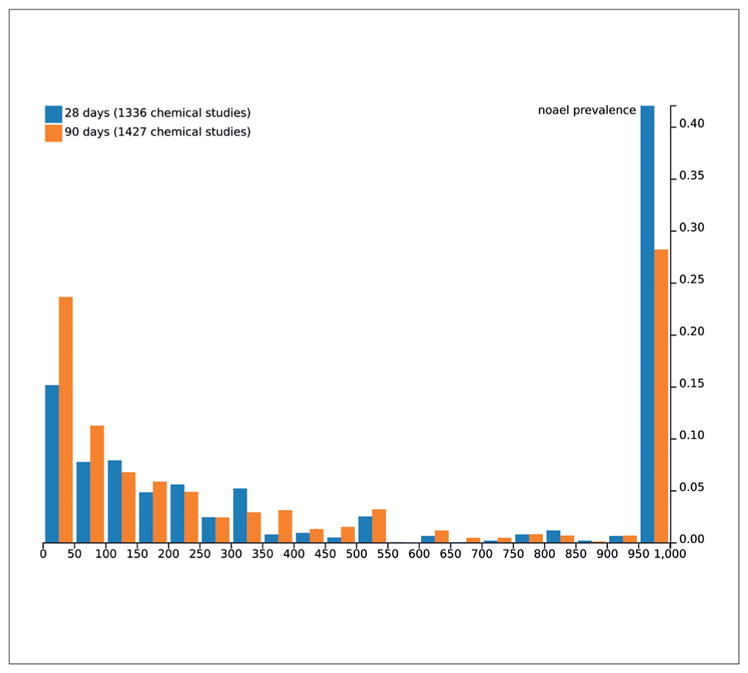
Prevalence frequency histogram of NOAELs for 28 and 90 day subchronic oral toxicity tests This figure was made by aggregating results of 2,400 90-day tests and 1,933 28-day tests.

Taylor et al.’s (2014) research suggests a redundancy between 28-day and 90-day repeated dose toxicity tests, proposing that the latter can be predicted given the former. They propose that non-toxicity (NOAEL ≥ 1,000 mg/kg b.w.) in the 28-day test is a strong predictor for non-toxicity in the 90-day study. 90-day tests are required by REACH for substances produced or marketed in a volume of 100 tons per year or above (Annex IX of Regulation (EC) No 1907/2006) (Aulmann and Pechacek, 2014). The assumption that a 90-day test is more sensitive than a 28-day test can be evaluated by observing the distribution of NOAEL’s for both tests independently and on the same substances. Using ECHA dossier study materials and methods data, we can test NOAEL prevalence by grouping all repeated dose oral toxicity tests with 28 day duration, and grouping tests with 90 day duration. Figure 3 shows that the 90-day test has a higher prevalence of low NOAEL’s than the 28-day test. Specifically, it appears that 90 day tests have a lower prevalence of NOAEL’s ≥ 1,000 mg/kg-day.

Chemical sets derived from 90-day and 28-day distributions in Figure 3 differ, with more substances having extractable 90-day studies than 28-day studies (1,427 vs. 1,336) and not all substances with 28-day studies having 90-day studies. This figure serves only as an indirect comparison of NOAEL distributions for the two tests.

Taylor et al. stipulate several requirements for test redundancy (Taylor et al., 2014):

- Experimental data equivalent to OECD TG 407, on the substance itself, conducted 1981 or later, with reliability score 1 or 2 and conducted to the limit dose (1,000 mg/kg b.w. per day) or higher, with a study result reported to be a NOAEL of 1,000 mg/kg b.w. per day or higher.
- The substance is not reported to be mutagenic or a skin sensitizer or acutely toxic by any route and there are adequate data to support this (i.e., any positive results from *in vitro* mutagenicity tests are followed up).
- There is no additional evidence based on physicochemical properties, structure or use to suggest that the substance could be biologically active.

**Fig. 4:**
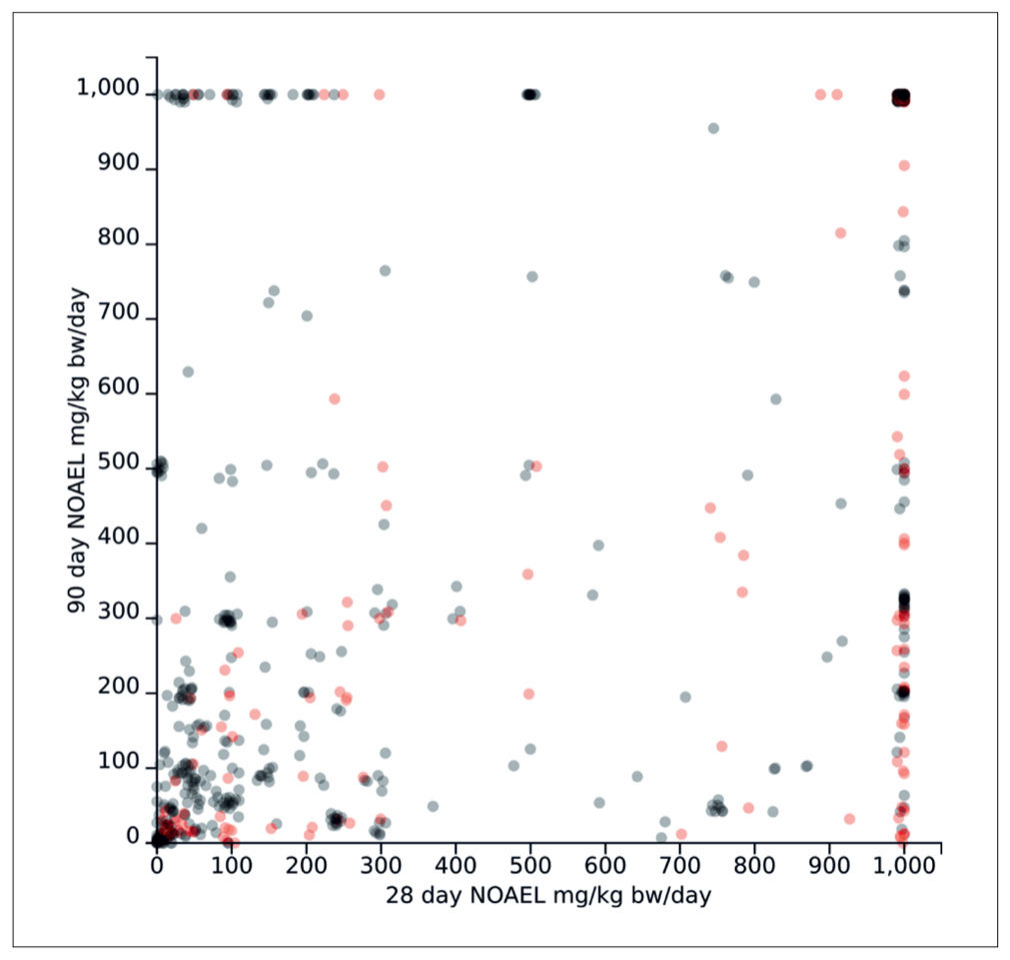
28- and 90-day acute oral toxicity matched NOAELs Circles represent the averaged 28 day (x-axis) and 90 day (y-axis) NOAEL for a given chemical taken from ECHA 28-day and 90-day oral toxicity studies. Red circles represent 200 substances matching the constraints given by Taylor et al. (2014).

In order to approximate these constraints, we required high reliability (Klimisch score of 1 or 2) and that substances have no positive GHS hazards for H300-H399. While this forms an imperfect reproduction, we found that all constraint meeting substances identified by Taylor (16 substances) were also identified in our query. Our query also identified an additional 184 substances meeting these constraints, making a total of 200 substances. Figure 4 visualizes Taylor constraint effects on the relationship between repeated dose 28-day and 90-day NOAELs for a set of 773 substances with data for both. Substances with both 28-day and 90-day NOAEL data matching the Taylor constraints are shown in red, substances failing to meet these constraints are in black. Several matching substances show higher 90-day NOAELS than 28-day NOAELs. We manually checked two of these substances:

- Ethoxy propoxy propanol (ECNumber 405-820-6) has one OECD TG 408 study (Repeated Dose 90-day Oral Toxicity) with a NOAEL of 1,000 mg/kg b.w. per day. It has a 28-day study with NOAELs of 225 mg/kg b.w. per day for female rats and 50 mg/kg b.w. per day for male rats. Large discrepancies between 28- and 90-day NOAELs may present study flagging opportunities, as it is unlikely for a 90-day study to report no toxicity when a 28-day study reports a low NOAEL. Such flags could identify potential faulty 28- or 90- day studies or other potential problems with a registration.
- Tris(2,4-ditert-butylphenyl) phosphite (EC Number 250-709-6) shows two 90-day studies reporting 500 mg/kg NOAEL for rats and > 318 mg/kg for dogs and one chronic study on rats with a NOAEL > 2,000 ppm (58-147 mg/kg b.w.). The single related 28-day study reports a 250 mg/kg b.w. per day NOAEL.

Taylor et al.’s primary hypothesis is that 28-day NOAELs greater than or equal to 1,000 mg/kg b.w. should predict high (≥ 1,000 mg/kg b.w. per day) 90-day NOAELs. This data point represents the upper right hand corner of Figure 4. We found that out of 121 substances meeting the Taylor constraints with 28-day NOAEL ≥ 1,000 mg/kg b.w., 70.2% also had 90-day NOAEL’s ≥1,000 mg/kg b.w., while the remaining 29.8% had lower 90-day NOAELs, which can be seen on the right side of Figure 4. In the total dataset (including substances not meeting Taylor constraints), 305 substances had 28-day studies with NOAEL ≥ 1,000 mg/kg b.w., with 68.9% having a matching high 90-day NOAEL and 31.1% having a lower NOAEL. This data shows that the 28-day NOAEL is predictive (albeit imperfectly) of 90-day NOAELs. However, the study constraints suggested by Taylor et al. do not appear to affect 28 day predictivity of the 90 day test, although we cannot rule out the possibility that this may be a consequence of a flawed constraint reproduction due to our reliance on machine readable values rather than manual curation of hundreds of studies.

The interrelationship of 28-day and 90-day NOAEL is also used very pragmatically in line with ECHA’s test guidance to estimate from a 28-day study a 90-day Derived No-Effect Level (DNEL) using an assessment factor of 3 (ECETOC, 2010). We used the dataset to test this assumption. 133 substances had both 9-day and 28-day key studies. Only, 11 chemicals had NOAEL below one third of the 28-day NOAEL, while 122 (91.7%) were within this limit. Given the reproducibility issues of repeated-dose studies (Wang and Gray, 2015), this is a strong confirmation of this pragmatic approach.

### 3.3 Acute oral toxicity modeling

#### 3.3.1 Dataset

A dataset of 1,059 substances was aggregated from the ECHA extraction for modeling acute oral toxicity. This modeling dataset consists of 268 positive (substances having an average reported LD_50_ in Klimisch score 1 studies of < 2,000 mg/ kg b.w. per day) and 791 negative substances. The Chemical Development Kit was used to generate 27 molecular descriptor values for each chemical in the dataset (Tab. 4). These descriptors were used in conjunction with substructure/Tanimoto-based k-nearest-neighbors (KNN) to make a similarity-informed multilayer perceptron. The evaluation metrics for KNN, decision tree and multilayer perceptron model are seen in Table 5.

**Fig. 5:**
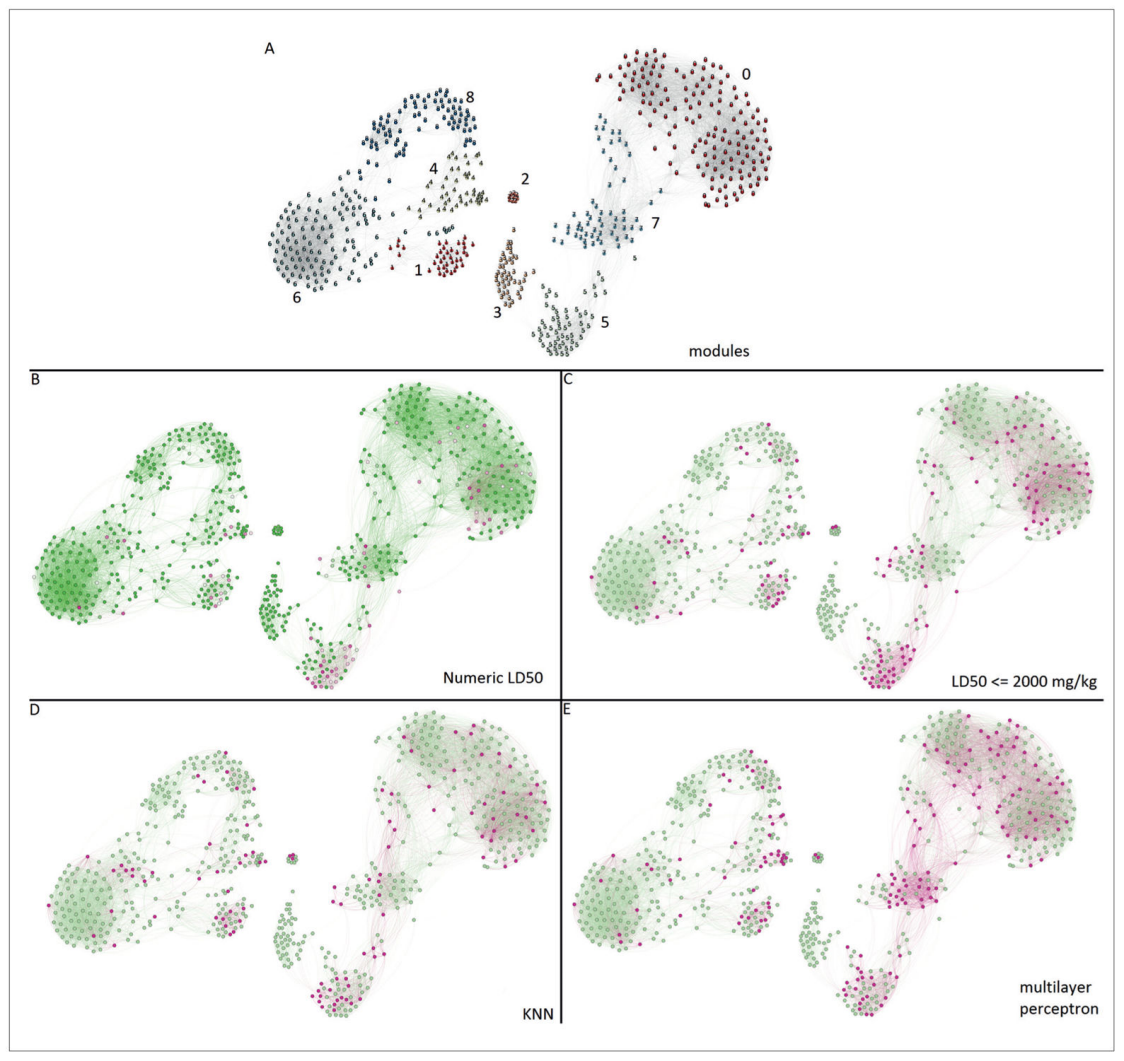
Chemical similarity maps for substances with acute oral toxicity LD_50_ data that could be mapped from REACH to PubChem The map contains 613 substances and is built from 3,122 substances mapped from REACH to PubChem and for which similarity and structure data could be determined from the chemistry development kit (Bolton et al., 2008; Steinbeck et al., 2003). Edges are shown between substances with similarity ≥ 0.7 as determined by their Tanimoto distance (Lourenço et al., 2004). The force layout algorithm is used to distribute substances (Fruchterman and Reingold, 1991). *A. Similarity map modules:* Nine modules are created by maximization of the Q-metric, a measure of module coherence (Blondel et al., 2008). Chemical nodes are colored by their module identification. *B. Chemical similarity map colored by experimental LD_50_*: Dark pink = low LD_50_, white = 1,000 mg/kg b.w./day, dark green = 2,000 mg/kg b.w./day. Results based on average LD_50_ values. Clusters of low LD_50_ values can be seen in module 5 and 0 with some otherwise sporadic distribution. *C. Chemical similarity map colored by oral toxicant status:* Pink substances denote LD_50_ < 2,000 mg/kg b.w. per day. Green substances denote LD_50_ ≥ 2,000 mg/kg b.w. per day (not classified). *D. KNN classifications for LD_50_*: Pink = predicted LD_50_ < 2,000 mg/kg b.w. per day. Green = predicted LD_50_ ≥ 2,000 mg/kg b.w. per day. Substances are predicted as toxicant if the majority of the closest 5 neighbors are toxicants. A chemical is considered a neighbor if it has Tanimoto similarity > 70% (Lourenço et al., 2004). *E. Multilayer perceptron classifications for LD_50_*: Pink = predicted LD_50_ < 2,000 mg/kg b.w. per day. Green = predicted LD_50_ ≥ 2,000 mg/kg b.w. per day. Classifier built on 1,059 substances referenced by at least one acute oral study with Klimisch score = 1. Classifications made by the multilayer perceptron appear to be well clustered; this indicates that chemical descriptors are influenced by substructure presence.

A chemical similarity map, Figure 5A, was created to explore the relationships between substances. The approach to the creation of this similarity map is given in more detail in (Luechtefeld et al., 2016, this issue). In short, edges are drawn between substances with high (> 70%) similarity as determined by Tanimoto distance. Similarity map layout was calculated via physical simulation by the force atlas algorithm (Jacomy et al., 2014; Bastian et al., 2009). The Blondel et al. (2008) modularity algorithm was applied to define the modules of the chemical similarity map. Figure 5A shows chemical modules which create an approach to the evaluation of model domains of applicability. Modules can be considered as having defining substructures, which are shared by the majority of module constituents. Module analysis for the ECHA-extracted substances was analyzed in greater depth in Luechtefeld et al.(2016, this issue). The LD_50_ of chemicals in this similarity map are visualized in Figure 5B for the 613 chemicals, which could be mapped to PubChem.

#### 3.3.2 K-nearest neighbors modeling

Evaluation of KNN on the global dataset results in a balanced accuracy of 71.4% with higher specificity than sensitivity (89.1% versus 53.7%) – a skew that is largely the consequence of the high prevalence of non-toxicants. The skew can also be attributed to using 2,000 mg/kg b.w. as a somewhat arbitrary threshold. Many chemicals come close to the 2,000 mg/kg b.w. threshold, which may make them more difficult classification targets. While traditionally seen as prevalence-independent, sensitivity and specificity of a machine learning algorithm such as KNN can be influenced by the balance of positive and negative substances than sensitivity in the training set. In this case, the large number of negative substances imbalances KNN towards a higher specificity.

The potential for KNN performance is suggested by visualizing clustering of toxicants in the chemical similarity map (Fig. 5C). The force atlas algorithm shows some strong clustering of oral toxicants, particularly in local regions of modules 3, 0 and 1. The impact of introducing the 2,000 mg/kg b.w. per day threshold can be seen by comparing Figure 5B and Figure 5C. Figure 5B is colored by LD_50_ value, whereas Figure 5C shows the binary classification. A particularly good example of the problems with selecting a threshold is seen in module 2 (a relatively non-toxic module), where two substances are defined as toxicants but can be seen to have high LD_50_ values (> 1,900 mg/kg b.w. per day) in Figure 5B.

The predictions made by KNN are visualized in Figure 5D. We can see that many of the toxicants shown in Figure 5C are missed; non-toxic neighbors of these toxicants are sometimes mislabeled as well. The naïve nature and relatively strong correspondence of toxicant locations provides motivation for the stronger instance-based learning models proposed by Jaworska and Nikolova-Jeliazkova (2007).

Table 6 gives the modular sensitivity and specificity for KNN. Several modules have relatively few oral toxicants (module 3 has 0 oral toxicants, module 2 has 2 oral toxicants) and thus care must be taken when evaluating on a per module basis. KNN is shown to perform more strongly for cohesive modules with their toxicants well clustered (module 5 vs. module 0). The overall sensitivity and specificity across all substances with a modularity class (615 substances) is 39.3% and 93.4%, representing a drop from the global sensitivity and specificity (53.7% and 89.1%).

#### 3.3.3 Supervised learning

Instance-based learning approaches derive strong “localized” results, but the lessons learned from substructures correlated with toxicity in one module cannot be extended to other modules by k-nearest neighbors. This fundamentally limits the KNN approach. Moreover, such an approach limits the understanding of toxic mechanisms. As some molecular and physical properties of substances are likely to be generally associated with toxicity status, we attempted to use a supervised learning model to derive rules from chemical features to predict toxicity. Such models essentially “learn” from large numbers of examples spanning the chemical universe. However, they fail to exploit relationships between substances on a local level. If a chemical is almost identical to an oral toxicant as determined via chemical structures, a supervised learning method only using molecular descriptors will fail to take advantage of this knowledge.

Instance-based learning methods can be used to inform supervised learning methods of chemical similarity data by constructing new features for supervised learning from the results of instance-based learning. Therefore, we used a toxic/non-toxic prediction from the KNN-model as a feature in a supervised learning model. The KNN feature ranks highest in both the Ranker and WrapperEval approaches to feature evaluation for acute oral toxicity. So while KNN alone does not adequately separate toxic from non-toxic substances, it contains more information than any chemical descriptor alone.

#### 3.3.4 Decision tree

To illustrate the combination of a KNN feature with chemical descriptors, we built a simple decision tree using the KNN feature and the next most informative feature, as determined by the Ranker approach to feature importance. In an attempt to balance the dataset, we weighted every toxicant in the dataset 2.95 times more heavily than non-toxicants; this makes the total weight of toxicants equal to non-toxicants with a total weight of 791 negative and 791 positive (268 before reweighting). This reweighting was done using the Weka toolkit (Hall et al., 2009). The resulting decision tree predicts that a chemical is positive when KNN is positive and negative when KNN is negative AND BPolDescriptor is > 10.95. Reweighting has the primary effect of improving the balance between sensitivity and specificity in the supervised learner trained on the reweighted data.

This tree has a resulting balanced accuracy of 70.4%, improved sensitivity (over KNN) of 68.7% and specificity of 71% (see Tab. 5). Interestingly, the second predictor – “BPolDescriptor” measures the sum of atomic polarizabilities of all bonded atoms in the molecule (Steinbeck et al., 2003); this descriptor may have simply separated out smaller substances with less labile bonds.

This decision tree illustrates how instance-based learning can be used in concert with supervised learning methods via feature generation. Although decision trees are fundamentally limited in their expressive power (they can only model endpoints via a conjunction of feature values), this model serves to demonstrate the possibility to improve sensitivity by fusing supervised and instance-based learning.

#### 3.3.5 Multilayer perceptron

Multilayer perceptron (feedforward artificial neural networks) algorithms can build more expressive feature relationships for toxicity prediction than decision trees. However, their resulting models are more difficult to visualize. Rather than only capturing conjunctions of feature values, multilayer perceptrons can model a wide variety of feature relationships. This “universal approximator” property is important for modeling potentially complex relationships of the sort chemical descriptors may have for predicting toxicological endpoints.

One consequence of the increased expression of multilayer perceptrons is the risk of overfitting. When training and testing multilayer perceptron on the entire dataset, the resulting sensitivity, specificity and balanced accuracy is 93.7%, 99.6% and 96.7%, respectively (Tab. 5). To account for overfitting, we performed stratified 10-fold cross-validation (after rebalancing instances by weighting toxicants 2.95x higher than non-toxicants). The stratified results show 70.7% sensitivity and 71.6% specificity for the multilayer perceptron (Tab. 5).

When evaluating feature importance we see that both the Ranker and WrapperEval approaches identify the KNN model feature as the most informative variable (Tab. 4). We use the KNN model as a feature for model training.

To visualize perceptron performance on each chemical in the chemical similarity map, we trained perceptrons on all other substances and predicted the chemical in question. This approach is referred to as leave-one-out cross-validation. The resulting perceptron prediction similarity graph (Fig. 5E) shows highly clustered toxicant predictions. This is perhaps a consequence of chemical descriptor dependency on substructures. In evaluating the leave-one-out perceptron on modules, we saw consistently higher sensitivity values relative to the KNN evaluation (Tab. 6 and 7) with an overall increased balanced accuracy of 69.4% resulting from significantly increased sensitivity and only slightly decreased specificity.

#### 3.3.6 Feature importance

Two approaches were taken to evaluate feature importance. For both approaches we split the dataset into 100 sub-datasets of equal size with balanced toxicant/non-toxicant prevalence.

In the first approach (Tab. 4), we evaluate the information gain of each feature across the sub-datasets. This approach is referred to as the Ranker approach (from its implementation in Weka.attributeSelection.Ranker; Hall et al., 2009).

In the second approach, we evaluate a classifier(decision tree classifier) by iteratively training on a training set comprised of 99 of the 100 subsets and testing on the remaining single dataset. Features were then removed and the impact on accuracy was ascertained. Features whose removal results in greater accuracy reduction show more promise to contribute to an effective set of features. This approach is referred to as the WrapperEval approach (from its implementation in Weka called WrapperSubsetEval; Hall et al. 2009). The instance-based learning KNN feature ranks highest in both ranker evaluation and wrapper evaluation indicating its independent and additive strength in predicting oral toxicity outcome.

The top three features as evaluated via independent oral toxicity information gain are KNN, Bpol, and AromaticBondsCount. Other than KNN, the wrapper evaluation approach to feature importance is not in strong agreement with the ranker approach. This may indicate redundancy between highly ranked ranker features such as KNN and Bpol. The wrapper evaluation approach is less sensitive to redundant, highly informative features.

KNN is selected in 100% of the 100 iterations of wrapper evaluation feature selection; topological polar surface area (TPSA) is selected in 84%. This indicates that TPSA and KNN represent some non-redundant information.

## 4 Discussion

Understanding limits of acute oral toxicity tests, LD_50_ biases of the various guidelines (due to dosing protocols), redundancy with other tests, and the potential of predictive algorithms to accurately classify a chemical can help improve and modernize acute toxicity guidelines. These data and algorithms can help experts to identify mechanisms behind toxicity via visualizations of decision trees, clustering of toxic/non-toxic chemicals with similar substructures and predictions of hazard for as yet untested chemicals.

In light of the ban on animal testing for cosmetic ingredients in Europe (Hartung, 2008) as well as the need to establish testing data for all tens of thousands of substances under REACH registration (Hartung, 2010), it is important to understand the practice of oral toxicity testing. The fact that consistency between guideline studies is in the 80% range shows against what an alternative approach should be measured.

While we found reasonable reproducibility amongst the various guidelines, both the moderately skewed probability of OECD TG 425 and the overall distribution of acute toxicity values cast some doubt on the usefulness of using a 2,000 mg/kg b.w. cut-off as a goal for modeling purposes. We found that a lack of toxicity in a 28-day study is a relatively good predictor that a chemical will be non-toxic acutely, but weaker support for the hypothesis that a 90-day dose can be extrapolated from a 28-day dose, and no support that further winnowing the data (i.e., by eliminating substances with hazard warnings) adds information for the purpose of extrapolating from short-term to long-term effects.

Going forward, a careful understanding of the value of each dosing regime – acute, subacute, subchronic and chronic – could likely guide a smarter testing regime tailored to each chemical class based on the likelihood of that test uncovering not just a NOAEL or a LOAEL but the most useful dose to inform hazard assessment. For example, integrated testing strategies can be built that use predictive models as an input into guideline protocols. There are many possible ways to integrate models into testing guidelines. One potential result would be to reduce animal use by starting dose protocols at higher levels for chemicals with low probabilities of toxicity.

Finally, our data indicate that while it is not straightforward to predict oral acute toxicity, it is not an intrinsically unsolvable problem. Overall, the KNN approach had relatively poor predictive power. However, our use of the simplistic PubChem 2D fingerprint is one source for this poor predictive power. Another explanation of the poor predictive power could be activity cliffs that limit the ability of QSAR models to predict acute toxicity over broad chemical classes. Our data indicates that it is likely a combination of those two explanations. Finally, the arbitrary choice of 2,000 mg/kg b.w. as a threshold for oral toxicant status may reduce modeling ability. It would appear that many of the substances that are likely non-toxic are within a range of LD_50_ values that are close enough to 2,000 mg/kg b.w. and thus the discretization of the data set into toxic/non-toxic at that dose likely complicates a model unnecessarily. More advanced instance-based learning models can avoid this problem by predicting actual NOAELs rather than binary thresholds. At the same time, it is clear that while acute oral toxicity largely does cluster together, there are certainly exceptions – in other words, significantly toxic compounds with structurally similar, non-toxic neighbors.

Although our models ultimately were not accurate enough to be useful from a hazard prediction perspective, our models were fairly naïve and worked purely off chemical descriptors. It is quite likely that additional descriptors – for example, corrosivity or sensitization – as well as a more detailed analysis of either ADMET or *in vitro* assays of biological activity – could substantially improve the models. The oral acute toxicity test has been subject to decades of refinement – reducing animals and reducing suffering. Our data suggest that the next “refinement” could come from the insights gleaned from a “Big Data” approach.

## Figures and Tables

**Tab. 1:** Five oral toxicity hazards extracted for 6,027 substances from ECHA dossiers “identification and labelling” information H300 has 36 failed extractions.

Description	Hazard	Positive	Negative*	Data lacking	Inconclusive
Fatal if swallowed	H300	33	5,709	237	12
Toxic if swallowed	H301	225	5,518	272	12
Harmful if swallowed	H302	1,072	4 677	266	12
May be harmful if swallowed	H303	23	5,720	272	12
May be fatal if swallowed and enters airways	H304	453	2,913	2,626	35
May be harmful if swallowed and enters airways	H305	3	3,316	2,628	35

* Negative refers to the ECHA “conclusive but not sufficient for classification” category.

**Tab. 2:** Percent agreement of OECD test guidelines for acute oral toxicity tests averaged over substances Number of substances with both tests in parentheses. Calculated by finding substances having studies with both guidelines where guidelines agree (defined as both having toxicity ≥ 2,000 or < 2,000 mg/kg b.w.) and dividing by total number of substances tested with both guidelines.

	OECD 401	OECD 420	OECD 423	OECD 425
**OECD 401**	93% (8,541)	90% (1,966)	74% (1,303)	83% (127)
**OECD 420**		92% (1,521)	81% (400)	92% (25)
**OECD 423**			90% (656)	84% (44)
**OECD 425**				94% (76)

**Tab. 3:** Evaluation of Bulgheroni et al. testing strategy on extracted ECHA data White cells only count experimental key *in vivo* oral toxicity studies. Grey cells count all oral toxicity studies (including read-across, and non-key studies).

NOAEL (mg/kg bw) from 28d study	LD_50_	(mg/kg bw)	Total
	< 2,000	≥ 2,000	
≤ 200	237	411	648
> 200	49	928	977
Total	286	1,339	1,625
≤ 200	660	1,301	1,961
> 200	183	3,126	3,309
Total	843	4,427	5,270

**Tab. 4:** Cross-validated feature importance via information gain, ranker evaluation and wrapper evaluation Summary descriptions of molecular descriptors used in perceptron training (as implemented by CDK).

Name	Description	Ranker Evaluation	Ranker stDev	Wrapper Evaluation
KNN (K=5,T=0.7)	K nearest neighbors with k=5 and neighbor threshold = 0.7	0.07	0.004	100
TPSA	Calculation of topological polar surface area based on fragment contributions	0.023	0.002	84
AcidicGroupCount	Returns the number of acidic groups	0	0	19
Apol	Sum of the atomic polarizabilities (including implicit hydrogens). Polarizabilities are taken from http://www.sunysccc.edu/academic/mst/ptable/p-table2.htm	0.05	0.003	15
HBondAcceptorCount	This descriptor calculates the number of hydrogen bond acceptors using a slightly simplified version of the PHACIR atom types	0.042	0.003	15
RuleOfFive	The number failures of Lipinski’s Rule of 5. See http://en.wikipedia.org/wiki/Lipinski%27s_Rule_of_Five	0.042	0.003	6
EccentricConnectivity	A topological descriptor combining distance and adjacency information	0.044	0.003	5
MannholdLogP	Prediction of logP based on the number of carbon and hetero atoms	0.041	0.003	4
AromaticAtomsCount	Number of aromatic atoms	0.008	0.005	4
Bpol	Sum of the absolute value of the difference between atomic polarizabilities of all bonded atoms in the molecule (including implicit hydrogens) with polarizabilities taken from http://www.sunysccc.edu/academic/mst/ptable/p-table2.htm	0.067	0.008	3
ZagrebIndex	The sum of the squares of atom degree over all heavy atoms i	0.044	0.003	3
FractionalPSA	Polar surface area expressed as a ratio to molecular size	0.004	0.009	3
LargestPiSystem	Number of atoms in the largest pi system	0.052	0.003	2
XLogP	Prediction of logP based on the atom-type method called XLogP	0.045	0.004	2
LargestChain	The number of atoms in the largest chain	0.047	0.006	1
HybridizationRatio	Reports the fraction of sp3 carbons to sp2 carbons	0.041	0.004	1
AromaticBondsCount	Number of aromatic atoms	0.067	0.005	0
RotatableBondsCount	The number of rotatable bonds is given by the SMARTS specified by Daylight on SMARTS tutorial	0.061	0.007	0
AtomCount	Number of atoms	0.054	0.007	0
FragmentComplexity	C=abs(B^2-A^2+A)+H/100 where C=complexity; A=number of non-hydrogen atoms; B=number of bonds and H=number of heteroatoms	0.054	0.004	0
Weight	Molecular weight	0.052	0.003	0
BondCount	Number of bonds of a given bond order (single, double, triple)	0.046	0.003	0
VadjMaDescriptor	Vertex adjacency information (magnitude): 1 + log2 m where m is the number of heavy-heavy bonds. If m is zero, then zero is returned (Definition from MOE tutorial on-line)	0.046	0.003	0
LongestAliphaticChain	Number of atoms in the longest aliphatic chain	0.045	0.008	0
PetitjeanNumber	Molecular graph descriptor measuring graph eccentricity	0.039	0.003	0
FMF	Ratio of heavy atoms in the Murcko framework to the total number of heavy atoms in the molecule	0.025	0.002	0
HBondDonorCount	Number of hydrogen bond donors using a slightly simplified version of the PHACIR atom types	0.02	0.004	0
BasicGroupCount	Returns the number of basic groups	0	0	0

**Tab. 5:** Sensitivity, specificity and balanced accuracy (BAC) of classifiers trained and tested on all substances existing in a module (see Fig. 5) Stratified 10-fold cross-validation was performed on the multilayer perceptron and indicates some overfitting of the decision tree.

Classifier	Train/Test	Sensitivity	Specificity	BAC
KNN (K=5,T=0.7)	1,059 substances, 268 positive 791 negative	53.7%	89.1%	71.4%
Decision tree*	1,059 substances, 268 positive 791 negative	68.7%	71.0%	69.9%
Multilayer perceptron*	1,059 substances, 268 positive 791 negative	93.7%	99.6%	96.7%
Multilayer perceptron*	stratified 10-fold cross validation	70.7%	71.6%	71.2%

* indicates that the classifier balanced positive and negative substances prior to training by weighting positive substances 2.95x heavier than negative examples, thus creating a balanced weight between negative and positive examples.

**Tab. 6:** Modular sensitivity and specificity of KNN These measurements give an idea of the domains of applicability for KNN and can be compared to the same domains in Table 8. KNN was not trained or tested in cross-validation but instead using the entire dataset for training and testing. Module 3 has no positive substances out of its 45 members making sensitivity not a number (NAN). BAC = balanced accuracy. FN = false negatives, TP = true positives, TN = true negatives, FP = false positives.

Module	Sensitivity	Specificity	BAC	FN	TP	TN	FP	Total
0	38.24%	94.83%	66.53%	21	13	110	6	150
1	50.00%	89.66%	69.83%	5	5	26	3	39
2	0.00%	85.71%	42.86%	2	0	6	1	9
3	NAN	100.00%	100.00%	0	0	45	0	45
4	0.00%	97.50%	48.75%	8	0	39	1	48
5	72.41%	55.17%	63.79%	8	21	16	13	58
6	20.00%	94.50%	57.25%	8	2	103	6	119
7	23.08%	93.75%	58.41%	10	3	45	3	61
8	0.00%	100.00%	50.00%	6	0	80	0	86
**ALL**	**39.29%**	**93.44%**	**66.36%**	**68**	**44**	**470**	**33**	**615**

**Tab. 7:** Modules sensitivity and specificity of multilayer perceptron trained in leave-one-out cross-validation Regions where molecular descriptors fail to predict oral toxicity accurately may be a consequence of the arbitrary threshold picked, or may indicate more subtle chemical effects not described by descriptors. FN = false negatives, TP = true positives, TN = true negatives, FP = false positives.

Module	Sensitivity	Specificity	BAC	FN	TP	TN	FP	Total
0	70.59%	69.83%	70.21%	10	24	81	35	150
1	60.00%	82.76%	71.38%	4	6	24	5	39
2	50.00%	85.71%	67.86%	1	1	6	1	9
3	NAN	100.00%	100.00%	0	0	45	0	45
4	37.50%	67.50%	52.50%	5	3	27	13	48
5	79.31%	41.38%	60.34%	6	23	12	17	58
6	20.00%	96.33%	58.17%	8	2	105	4	119
7	61.54%	45.83%	53.69%	5	8	22	26	61
8	0.00%	93.75%	46.88%	6	0	75	5	86
**ALL**	**59.82%**	**78.93%**	**69.37%**	**45**	**67**	**397**	**106**	**615**

## Footnotes

1 http://wapo.st/1KpRRiH

2 http://bit.ly/1nMMr7B

3 http://www.unece.org/fileadmin/DAM/trans/danger/publi/ghs/ghs_rev02/English/03e_part3.pdf
